# Aminoflavone upregulates putative tumor suppressor miR-125b-2-3p to inhibit luminal A breast cancer stem cell-like properties

**DOI:** 10.1093/pcmedi/pbac008

**Published:** 2022-03-28

**Authors:** Nicole Mavingire, Petreena Campbell, Tiantian Liu, Jonathan Wooten, Salma Khan, Xin Chen, Jason Matthews, Charles Wang, Eileen Brantley

**Affiliations:** Department of Basic Sciences, Loma Linda University School of Medicine, Loma Linda, CA 92350, USA; Department of Basic Sciences, Loma Linda University School of Medicine, Loma Linda, CA 92350, USA; Current address: Frederick National Laboratory for Cancer Research, PO Box B, Bldg. 432, Room 232 Frederick, MD 21702-1201, USA; Department of Basic Sciences, Loma Linda University School of Medicine, Loma Linda, CA 92350, USA; Center for Genomics, Loma Linda University School of Medicine, Loma Linda, CA 92350, USA; Department of Basic Sciences, Loma Linda University School of Medicine, Loma Linda, CA 92350, USA; Center for Health Disparities and Molecular Medicine, Loma Linda University School of Medicine, Loma Linda, CA 92350, USA; Department of Basic Sciences, Loma Linda University School of Medicine, Loma Linda, CA 92350, USA; Center for Health Disparities and Molecular Medicine, Loma Linda University School of Medicine, Loma Linda, CA 92350, USA; Department of Basic Sciences, Loma Linda University School of Medicine, Loma Linda, CA 92350, USA; Center for Genomics, Loma Linda University School of Medicine, Loma Linda, CA 92350, USA; Department of Nutrition, University of Oslo, Oslo 0372, Norway; Department of Pharmacology and Toxicology, University of Toronto, Toronto, Ontario M5S 1A8, Canada; Department of Basic Sciences, Loma Linda University School of Medicine, Loma Linda, CA 92350, USA; Center for Genomics, Loma Linda University School of Medicine, Loma Linda, CA 92350, USA; Department of Basic Sciences, Loma Linda University School of Medicine, Loma Linda, CA 92350, USA; Center for Health Disparities and Molecular Medicine, Loma Linda University School of Medicine, Loma Linda, CA 92350, USA

**Keywords:** breast cancer, cancer stem cells, therapeutic targeting, miR125b-2–3p, aminoflavone, aryl hydrocarbon receptor, mammospheres

## Abstract

Metastatic breast cancer is incurable and often due to breast cancer stem cell (CSC)-mediated self-renewal. We previously determined that the aryl hydrocarbon receptor (AhR) agonist aminoflavone (AF) inhibits the expression of the CSC biomarker α6-integrin (ITGA6) to disrupt the formation of luminal (hormone receptor-positive) mammospheres (3D breast cancer spheroids). In this study, we performed miRNA-sequencing analysis of luminal A MCF-7 mammospheres treated with AF to gain further insight into the mechanism of AF-mediated anti-cancer and anti-breast CSC activity. AF significantly induced the expression of >70 microRNAs (miRNAs) including miR125b-2–3p, a predicted stemness gene regulator. AF-mediated miR125b-2–3p induction was validated in MCF-7 mammospheres and cells. miR125b-2–3p levels were low in breast cancer tissues irrespective of subtype compared to normal breast tissues. While miR125b-2–3p levels were low in MCF-7 cells, they were much lower in AHR^100^ cells (MCF-7 cells made unresponsive to AhR agonists). The miR125b-2–3p mimic decreased, while the antagomiR125b-2–3p increased the expression of stemness genes ITGA6 and SOX2 in MCF-7 cells. In MCF-7 mammospheres, the miR125b-2–3p mimic decreased only ITGA6 expression although the antagomiR125b-2–3p increased ITGA6, SOX2 and MYC expression. AntagomiR125b-2–3p reversed AF-mediated suppression of ITGA6. The miR125b-2–3p mimic decreased proliferation, migration, and mammosphere formation while the antagomiR125b-2–3p increased proliferation and mammosphere formation in MCF-7 cells. The miR125b-2–3p mimic also inhibited proliferation, mammosphere formation, and migration in AHR100 cells. AF induced AhR- and miR125b2-3p-dependent anti-proliferation, anti-migration, and mammosphere disruption in MCF-7 cells. Our findings suggest that miR125b-2–3p is a tumor suppressor and AF upregulates miR125b-2–3p to disrupt mammospheres via mechanisms that rely at least partially on AhR in luminal A breast cancer cells.

## Graphical Abstract

**Graphical Abstract:**
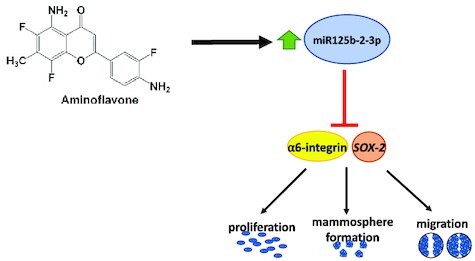

## Introduction

Breast cancer (BC) is the most diagnosed cancer in women and the second leading cause of cancer death in women in the USA.^1,2^ Endocrine therapy (ET) is the most common treatment modality for BC patients with tumors that express hormone receptors (luminal subtype) and is initially quite effective. However, up to 40% of patients with luminal BC will experience ET resistance, which often leads to recurrence, metastasis, and death.^3^ This includes those with the luminal A subtype, which is associated with a better prognosis than the luminal B subtype due to a lower proliferative index. Breast cancer stem cells (CSCs) contribute to the development of ET resistance because of their ability to evade detection, migrate, invade other sites, and self-renew to produce tumors that fail to respond to ET.^4^ CSCs also contain unique molecular aberrations that promote ET resistance, drive metastasis, and promote relapse.^5–7^ Therefore, targeting and eliminating CSCs is crucial to counteracting ET resistance.

Stemness genes, including those that regulate certain integrins, have been linked to drug resistance and disease progression.^8^ α6-Integrin is a putative stemness biomarker expressed in several solid cancers.^9,10^ High α6-integrin expression is linked to breast tumor initiation and decreased patient survival.^11^ Furthermore, BC tissues from patients who relapsed on tamoxifen (an ET agent), show elevated α6-integrin expression compared to treatment-naïve tissues.^12^ Mammospheres, which enrich for CSCs, overexpress α6-integrin,^13^ while α6-integrin knockdown disrupts mammospheres and eliminates mammosphere-mediated tumorigenicity when transplanted into mice.^9^ We previously determined that aminoflavone (AF) disrupts tamoxifen-resistant (TamR) mammospheres, and inhibits TamR cell growth, α6-integrin expression, and α6-integrin-Src-Akt signaling activation.^12,13^ AF activates aryl hydrocarbon receptor (AhR) signaling and is thus considered to be an AhR agonist.^14^ Historically, AhR agonists consisted of environmental toxins, and their activation of AhR signaling led to the formation of carcinogenic metabolites.^14^ More recently, AhR agonists have been shown to demonstrate activity against BC thus revealing the AhR as a viable therapeutic target.^14–16^ We and others have recently shown that AhR agonists such as AF disrupt mammospheres in part by inhibiting stemness gene expression.^17–19^ AhR ligands also modulate microRNA (miRNA) expression in BC to promote anticancer actions or reverse drug resistance.^18^

miRNAs are short (18–28 nucleotides long) non-coding RNAs that silence gene expression post-transcriptionally by binding to target mRNAs to thwart their translation or to promote their degradation.^20^ Aberrantly expressed miRNAs have been increasingly implicated in promoting BC progression and drug resistance.^21^ Additionally, multiple miRNAs have been shown to play a role in ET resistance.^21,22^ The miR125 family plays a variety of roles depending on the cellular context, as reviewed by Sun *et al*.^23^ In BC, miR125 family members are primarily tumor-suppressive by regulating various signaling pathways that control cell proliferation, epithelial-to-mesenchymal transition (EMT), differentiation, metabolism, and tumor immunity in receptor tyrosine-protein kinase erbB-2-overexpressing and triple-negative BC. miR125a and miR125b mediate the erbB2/Her2 and NFκB pathways, target the oncoprotein and transcription factor ETS1, and target and suppress the expression of oncoprotein mucin 1 to promote DNA damage-induced apoptosis and cell-cycle arrest in BC cells.^24,25^ Furthermore, pre-miR125b has been shown to decrease tumor weight and volume of triple-negative BC and melanoma in mouse models.^25^ miR-125a, cooperates with miR-125b and miR-205 to down-regulate erbB2/erbB3 and promote apoptosis in BC cells.^26^ miR-125a suppresses proliferation and tumor progression by down-regulating BRCA1-associated protein 1 (BAP1).^27^ miR-125b-5p inhibits proliferation, migration, and invasion by targeting KIAA1522 in BC cells.^28^ miRNA sequencing data reveal that miR-125a and miR-125b are down-regulated in biopsy specimens from breast and prostate cancer patients.^29,30^ Microarray data showed that miRNA profiles that include miR125a and miR125b could stratify BC by receptor subtype.^31^ Notably, the greatest miR125b up-regulation occurred in erbB2-negative samples vs erbB2-positive ones.^31^ Sun *et al*. analyzed 175 pairs of BC and normal control samples over 80 months and found that miR125b highly regulates tissue-specific transplantation antigen P35B (TSTA3), which is important prognostically because TSTA3 is highly expressed in BC tissues and tumor cells, such that expression is closely related to cancer stage. Furthermore, patients with tumor overexpressing TSTA3 have low survival rates.^32^ Additionally, miR125b regulates cell survival, proliferation, and invasion via the ErbB2/Her2 pathway in breast and endometrial cancers.^33,34^ Most recently, Incoronato *et al*. were able to differentiate between BC patients and normal healthy patients based on miR125b levels in their blood.^35^ Taken together, miR125b has the potential to function as both a druggable target and prognostic marker in BC patients across all subtypes. However, miR125b in earlier literature refers to miR125b-5p under current nomenclature.^36,37^

miR125b-5p and miR125b-2–3p originate from the same miRNA stem-loop MIR125B2 on chromosome 21.^38^ miR125b-2 has also been mapped as part of the MIR99A-MIRLET7C-MIR125B2 cluster on chromosome 21.^39^ While a recent publication suggests that miR125b-2 possesses potential tumor suppressor actions in gastric cancer^40^, its role in luminal BC remains undefined. Two recent publications identified miR125b-2 as part of a panel of miRNAs that indicate excellent diagnostic performance in BC.^41,42^ Notably, both research groups found that miR125b-2 expression is down-regulated in luminal BC tissues compared to normal breast tissues.^41,42^ We, therefore, sought to delineate the role of miR125b-2–3p in luminal A BC cell lines MCF-7 and AHR^100^ and to determine whether AF regulates miR125b-2–3p to confer its anticancer actions and thwart CSC activity in BC cells and mammospheres. Lastly, we sought to delineate the role of AhR in mediating the anticancer and anti-CSC actions of miR125b-2–3p in breast cancer cells and mammospheres.

## Materials and methods

### Cell culture and reagents

The luminal A breast cancer subtype cell line human MCF‐7 (ATCC® HTB-22™) was obtained from the American Type Culture Collection (ATCC) and maintained as previously described.^12^ The normal non-malignant breast epithelial cell line MCF-10A (ATCC® CRL-10317^™^) was obtained from the ATCC and maintained per the manufacturer's handling instructions. AhR-deficient MCF-7 (AHR^100^) cells were generated from wild-type (WT) MCF-7 cells by continuous exposure for 6–9 months to 100 nmol/L benzo(a)pyrene.^43^ These cells exhibit a 100-fold higher resistance to benzo(a)pyrene than do the wild-type MCF-7 cells. The AHR^100^ cells had no detectable amounts of the AhR and normal concentrations of aryl hydrocarbon nuclear translocator^44^. MCF‐7 and AHR^100^ breast cancer cells were cultured in RPMI‐1640 medium containing 10% fetal bovine serum (FBS) (Hyclone, Logan, UT), supplemented with 2 mM glutamine and penicillin and streptomycin antibiotics (Mediatech, Herndon, VA). MCF-7ZFn AhR-knockout (AHRKO) cells were generated by previously described methods.^45^ Briefly, validated CompoZr knockout ZFN plasmids targeting AHR were transfected into MCF-7 cells followed by several serial dilutions and clone selection. Clones were screened for the presence of indels at the ZFN recognition site in exon 1 of AHR by DNA sequencing, and AhR knockout was confirmed by assessing downstream targets of AhR (e.g., CYP1A1) with real-time quantitative polymerase chain reaction (qPCR). AHRKO cells and the corresponding wildtype cells (MCF-7 WT) were cultured in Dulbecco's Modified Eagle's Medium (DMEM) supplemented with 10% (vol/vol) FBS and 1% penicillin/streptomycin (PS). 5‐Amino‐2‐(4‐amino‐3‐fluorophenyl)‐6,8‐difluoro‐7‐methyl‐4H‐1‐benzopyran‐4‐one (AF) was obtained from The NCI/DTP Open Chemical Repository (http://dtp.cancer.gov, Frederick, MD) at the Frederick National Laboratory for Cancer Research. Stock solutions of AF were dissolved in dimethyl sulfoxide (DMSO). All stocks were stored protected from light at −20°C until use. Treatments involving DMSO used no more than 0.1% so as to not interfere with cell behavior.

### Tumor specimens and RNA extraction

Fourteen breast tumor specimens were retrieved from patients who relapsed on ET in accordance with an Institutional Review Board (IRB) approved protocol from the Loma Linda University ethics committee. Three of the patients experienced relapse following treatment with ET. All patients provided informed consent. Formalin-fixed paraffin embedded (FFPE) tissues were cut into 4 μm sections and miRNA was isolated from FFPE breast cancer tissue specimen slides using the miRNeasy FFPE Kit (Qiagen, Germantown, MD) following the manufacturer's instructions. Briefly, FFPE cancer tissue sections were deparaffinized with xylene treatment followed immediately by a 100% ethanol wash. Protein was degraded with proteinase K, and DNA was degraded by DNAse I. RNA was purified with buffer washes then eluted in nuclease-free water and stored in a −80°C freezer until use.

### RNA extraction, quantitative reverse transcription-polymerase chain reaction analysis

Total RNA was isolated from MCF-10A, MCF‐7, and AHR^100^ cells (grown in monolayers) or as mammospheres using the miRNeasy Mini kit (Qiagen, Germantown, MD) following the manufacturer's instructions. miRNA was also isolated from five FFPE breast cancer tissue specimen slides (seeTumor specimens and RNA extraction section). Complementary DNA (cDNA) was prepared using an iScript Advanced cDNA synthesis kit (Bio‐Rad, Richmond, CA) or the miScript II RT Kit (Qiagen, Germantown, MD), or the miRCURY LNA RT Kit (Qiagen, Germantown, MD). Quantitative real‐time polymerase chain reaction (PCR) analysis was performed using Power SYBRGreen supermix (Thermo Fisher Scientific, Rockford, IL), or the miScript SYBR® Green PCR Kit (Qiagen, Germantown, MD), or the miRCURY LNA SYBR® Green PCR Kit (Qiagen, Germantown, MD). glyceraldehyde-3-phosphate dehydrogenase (GAPDH) (PCR) or SNORD95-11 (miScript RNA PCR) and SNORD44 (miRCURY RNA PCR) small nuclear RNA was used as an endogenous control for normalization. ITGA6, SOX2, MYC, and miR125b-2–3p reverse transcription was performed using 1 μg of total RNA from each sample. A CFX‐96 or CFX-96 Touch PCR thermocycler instrument (Bio‐Rad, Hercules, CA) was used to generate PCR products. PCR products were obtained using reagents from Qiagen (Germantown, MD). When making comparisons among treatment groups across cell lines, gene expression was normalized to DMSO for the same cell line. No normalizations across different cell lines were made due to variations in reference gene expression between cell lines.

### miRNA sequencing and bioinformatics analysis

#### miRNA sequencing

miRNA-seq libraries for MCF-7 mammospheres (three for AF treated and three for DMSO treated) were constructed using the QIAseq miRNA library (Qiagen, Hilden, Germany) kit following the manufacturer's instructions. Briefly, 3′ and 5′ adaptors along with unique molecular identifiers (UMI) were added to small RNAs. Reverse transcription was performed to convert the target small miRNAs into cDNAs. 22-Cycles of PCR amplification was performed. An assigned index was given to each sample for the multiplexing. After size selection by magnetic beads, DNA fragments with the correct insert sizes were selected for the miRNA-seq library. Libraries were quantified by the Qubit 3.0 HS dsDNA assay (Thermal Fisher Scientific, Waltham, USA). Library size and quality were examined using the TapeStation 2200 (Agilent, Santa Clara, USA). 76 bp single-end sequencing was performed on Illumina HiSeq 4000.

#### miRNA-seq data analysis

All sequencing data were demultiplexed and converted to fastq files using bcl2fastq (Illumina Inc., San Diego, USA). After quality checking with FastQC tool, the fastq files were processed using the QIAseq miRNA quantification feature provided by Qiagen GeneGlobe Data Analysis Center (Qiagen). Briefly, reads were trimmed of 3′ adaptor and the low-quality bases using Cutadapt. After trimming, reads shorter than 16 bp and <10 UMI counts were excluded. Alignment was performed using bowtie with a maximum of two mismatches tolerated. Aligned reads were annotated using miRBase V21. Differentially expressed miRNAs were identified by R package ‘DESeq2’ using DMSO-treated cell lines as a control group. Duplicate removed UMI counts were used for differential expression analysis. miRNAs with false discovery rate (FDR) adjusted *P*-value <0.05 and absolute log2 (fold-change) >0.5 were considered significantly differentially expressed. The top 30 variant genes heatmap was generated using the R package ‘pheatmap’. A volcano plot was generated using the R package ‘ggplot2’. miRNA target prediction was performed using Ingenuity Pathway Analysis (IPA, Ingenuity Systems, Redwood City, CA) microRNA Target Filter. Only experimentally observed gene targets based on four public databases (TargetScan, TarBase, miRecords, and Ingenuity Knowledge Base) were included in subsequent analysis. Go term enrichments of predicted target genes were performed using the R package ‘ClusterProfiler’.

### Western blotting

MCF-7 and AHR^100^ cells were seeded at 3 × 10^6^ per dish (100 mm) and serum-starved for 24 h. After 24 h, cells were treated with medium containing DMSO or 1 μM AF for 48 h. For AhR-inhibition experiments, cells were treated with medium containing 100 nM α-naphthoflavone (αNF) for 1 h before treatment with DMSO or AF for 48 h. After the treatment, cells were harvested via scraping on ice, protein was extracted, and western blot analysis was performed as described.^15^ Briefly, proteins were resolved on 4%–12% sodium dodecyl sulfate polyacrylamide gels and transferred to nitrocellulose membranes. The membranes were blocked before overnight incubation at 4°C in 5% milk–based buffer with rabbit primary antibodies against α6-integrin (1:1000) and rabbit primary antibodies against β-actin (1:1000) (Cell Signaling Technology, Danvers, MA). Membranes were incubated with the appropriate horseradish peroxidase-conjugated secondary antibodies (Cell Signaling Technology, Danvers, MA) for 1 h at the appropriate dilution before imaging. 1D-anaylsis was completed using analytikjena UVPChemStudio instrument (Germany) and α6-integrin expression was normalized to β-actin expression.

### Silencing and/or overexpressing by 2′deoxy, 2′fluroarabino nucleic acids

The silencing and/or overexpressing of miR125b-2–3p in MCF-7 and AHR100 cells was achieved by 2′deoxy, 2′fluroarabino nucleic acid (2′F-ANA)-modified oligonucleotides (ASOs) purchased from AUM Biotech (Philadelphia, PA, USA). The sequence of miR125b-2–3p is ucacaagucaggcucuugggac. Briefly, for silencing experiments, 5 × 10^5^ cells were plated in complete medium overnight in 6-well plates and then treated with either 0.70 µM 2′F-ANAs against miR125b-2–3p or a SCRAMBLE 2′F-ANA for 24, 48, and 72 h. To overexpress miR125b-2–3p, 5 × 10^5^ cells were plated in complete medium overnight in 6-well plates and then treated with either 0.75 µM 2′F-ANA mimics to miR125b-2–3p or a SCRAMBLE 2′F-ANA for 24, 48, and 72 h. Silencing/overexpressing efficiency was analyzed by quantitative reverse transcription-PCR. AntagomiR/mimic-treated cells were then replated after 48 h for colony formation, mammosphere formation, or wound healing experiments. Results presented in this study are based on 48 h antagomiR/mimic exposure.

### Mammosphere formation assay

Cells were cultured in suspension as mammospheres using the MammoCult™ human medium kit (Stem Cell Technologies, Vancouver, BC). MammoCult™ medium consists of mammary epithelial growth medium (MEGM), supplemented with B27, 20 ng/mL epidermal growth factor (EGF), 20 ng/mL bFGF, and 4 μg/mL heparin. Bovine pituitary extract was excluded.^46^ Mammospheres were cultured for 3 days in Falcon 6‐well nontreated polystyrene plates (product #351146 FisherScientific, Tustin, CA) before being exposed to the respective treatments for 48 h. Mammospheres were visualized using an IX‐71 Olympus microscope (Hoffman modulation contrast mode, Olympus Life Sciences Solutions, Waltham, MA) and pictures were taken before and after treatment. Additionally, mammospheres were collected and prepared for qPCR analysis as described previously.^13^

### Colony formation assay

MCF-7 and AHR^100^ cells were plated at a density of 2000 cells per well in NUNC six‐well plates (Thermo Fisher Scientific, Rockford, IL) and treated with either DMSO or AF for 24 h, then allowed to grow for 2 weeks before the cells were fixed with 10% formalin and stained with crystal violet solution. Colonies were imaged and counted using the open-source image processing software ImageJ (National Institutes of Health, Bethesda, MD) for colony formation analysis. Use of this software enabled a non-biased assessment of the colony number.

### Wound healing assay

MCF-7, AHR^100^, MCF-7-WT, and AHRKO cells were plated in NUNC 24‐well plates (Thermo Fisher Scientific, Rockford, IL) using the IBIDI Culture-Inserts 2 well for self-insertion (Gräfelfing, Germany) following the manufacturers’ instructions for the wound healing assay. Cells could recover over 24 h before inserts were removed using tweezers. The cells were then treated with 0.1% DMSO or 10 nM, 100 nM, or 1µM AF for 48 h. Images were captured on an Olympus IX‐71 microscope and quantified using SPOT software (Olympus Life Sciences Solutions, Waltham, MA) or the open-source image processing software ImageJ analysis software (National Institutes of Health, Bethesda, MD). Migration rate was calculated as the size of the scratch at 48 h minus the size of the scratch at 0 h, divided by 48 h. Migration rates for each treatment were then subtracted from the migration rate for DMSO treatment to yield the relative cell migration rate.

### Statistical analysis

Differences between multiple groups were analyzed using one‐way analysis of variance (ANOVA) with Tukey's test or the Tukey–Kramer multiple comparison tests for evaluating three or more groups. To compare the two groups, the unpaired Student's t-test was used, with Welch's correction only when the standard deviations between the two groups were very different. Statistical analysis was performed using GraphPad Prism 9.2 (GraphPad Software, Inc., San Diego, CA; www.graphpad.com). Differences were considered significant at *P* < 0.05.

## Results

### Aminoflavone induced miR125b-2-3p expression in MCF-7 cells and mammospheres

**Figure 1.**
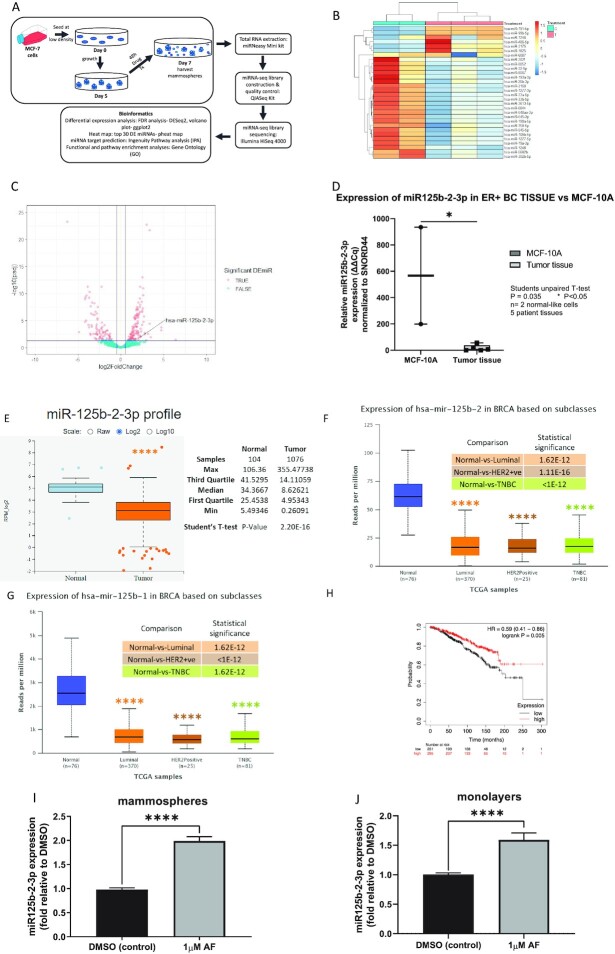
Aminoflavone modulates miRNAs in MCF-7 mammospheres while miR125b-2–3p expression is low in breast cancer tissue. (**A**) Next-generation sequencing study design. Mammosphere samples were collected. Total RNA was extracted and miRNA-seq libraries were constructed. QC analysis and quantification were performed in all miRNA-seq libraries before sequencing. After sequencing, the reads were aligned and counted using QIASeq miRNA sequencing pipeline. miRNA target prediction analysis was conducted using the differentially expressed miRNAs identified. (**B**) Heatmap displays the top 30 most variant miRNAs in treated and control groups and the hierarchical clustering of all samples. The color scale is in increasing order of Z-Score from blue to red. Z-Score is calculated based on DESeq2 rlog transformed miRNA read counts. (**C**) Volcano plot showing differentially expressed miRNAs between treated and control groups. The *y*-axis represents the -log10padj and the *x*-axis displays the log2 (fold-change) value. A positive *x*-value represents an up-regulation, and a negative *x*-value represents a down-regulation. –log10 (padj) >1.3 (padj < 0.05) and absolute log2 (fold-change) > 0.5 were marked as the significance threshold. Each dot represents one differentially expressed miRNA, with those above the significance threshold are highlighted in red. miR-125b-2–3p is highlighted in the plot. (**D**) RT-qPCR analysis of miR125b-2–3p expression in ER + BC samples compared to MCF-10A cells. BRCA, breast cancer; HER-2, human epidermal growth factor receptor 2; TNBC, triple negative breast cancer. (**E**) mir-tv analysis of miR125b-2–3p expression in BC samples in the TCGA database. (**F, G**) UALCAN analysis of miR125b-1 and miR125b-2 expression in BC samples in the publicly accessible TCGA database stratified by subclass. (**H**) Survival curves based on Metabric analysis of overall survival predicted among patients with luminal A breast cancer concerning miR125b tumor expression levels. *P*-value = log-rank test, HR hazard ratio. (**I**) Real-time qPCR analysis of miR125b-2–3p expression in MCF-7 mammospheres, and (**J**) cells after 48 h treatment with either DMSO or 1 μM AF. ^****^P < 0.0001, compared to DMSO.

Next-generation sequencing (miRNA sequencing) was used to detect the expression profile of miRNAs in MCF-7 mammospheres exposed to either DMSO (vehicle control) or AF for 48 h (Fig. 1A). An average of 4.6 million reads were mapped to mature miRNA for each library. Bioinformatics analysis revealed that 2039 miRNAs were detected in all samples. A total of 206 miRNAs were identified as significantly differentially expressed miRNAs (DEmiRNAs) between the control group (0.1% DMSO) and the treatment group (AF, 1 µM), with FDR adjusted *P*-value < 0.05 and absolute log2 (fold-change) > 0.5 (Supplementary Table I, see online supplementary material).

Among 152 certain DEmiRNAs, 64 were down-regulated and 88 significantly up-regulated in mammospheres exposed to AF (*P* adjusted < 0.01). The top 30 most variant miRNAs across all samples are shown in the heat map in Fig. 1B. The full list of certain up-regulated and down-regulated miRNAs is provided in Supplementary Table II, see online supplementary material. The volcano plot reveals differential miRNA expression in mammospheres exposed to AF as compared to vehicle control for statistical significance (Fig. 1C).

**Figure 2.**
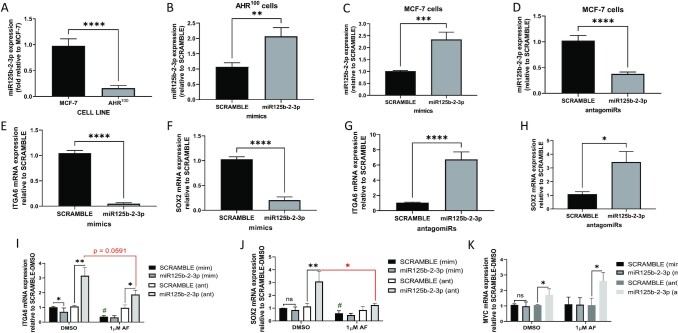
miR125b-2–3p suppresses ITGA6 and SOX2 mRNA expression in breast cancer cells while aminoflavone inhibits ITGA6 as well as SOX2 mRNA expression in mammospheres. Real-time qPCR analysis of miR125b-2–3p basal expression in (**A**) AHR^100^ vs MCF-7 cells, (**B**) miR125b-2–3p expression after 48 h mimic treatment in AHR^100^ cells, (**C**) miR125b-2–3p expression after 48 h mimic treatment in MCF-7 cells, (**D**) miR125b-2–3p expression after 48 h antagomiR125-b-2 treatment in MCF-7 cells. qPCR analysis of mRNA expression in MCF-7 cells exposed to either miR125b-2–3p mimics (**E** and **F**), or antagomiRs (**G** and **H**) or scramble for 48 h. qPCR analysis of mRNA expression in MCF-7 mammospheres exposed (**I**–**K**) to either miR125b-2–3p mimic, or antagomiR125b-2–3p, or scramble controls followed by 48 h AF treatment. ^#^*P* < 0.05, ^****^P < 0.0001, ***P < 0.001 and **P < 0.01, *P < 0.05 compared to control and ns indicates not significant. Columns, mean of three independent experiments: error bars, SEM.

In Supplementary Table I, three miR125 members are highlighted in bold because, as outlined in the introduction, these are the miRNAs we were most interested in. The miR125 family plays a variety of roles depending on the cellular context.^23^ Subsequently, we narrowed our focus to miR125b-2–3p for multiple reasons. Firstly, while all three miR125 members on the DEmiRNA list were significantly upregulated in mammospheres after AF treatment, miR125b-2–3p was the second most upregulated of the three (padj = 0.017, log2 (fold-change) = 2.07). Secondly, the IPA miRNA target analysis identified 859 experimentally verified mRNAs targeted by the significant DEmiRNAs in the AF-treated group compared with the control group (Supplementary Table III, see online supplementary material). In total, 81 of these genes have been experimentally verified as targets of miR125b-5p (highlighted in yellow, Supplementary Table III), but experimentally verified targets of miR125b-2–3p are yet to be determined. This points to a gap in our knowledge of miR125b-2–3p's role in gene regulation and BC. To our knowledge, we are the first to experimentally investigate three potential target genes of miR125b-2–3p. Our in-house RT-qPCR analysis using 5 ER + BC FFPE tissue samples showed that miR125b-2–3p expression was lower in tumor tissues when compared to the expression in non-tumorigenic breast epithelial MCF-10A cells (Fig. 2D). Of note, mir-tv analysis of TCGA data showed that miR125b-2–3p is significantly decreased in non-stratified BC tissue samples vs normal tissues (Fig. 2E).^47^ Using the UALCAN tool for analyzing omics data in the TCGA database, we found that the expressions of both miR125b2 (the miRNA loop that the miR125b-2–3p arm comes from) and the miR125b1 loop were significantly downregulated in BC tissues compared to normal tissues irrespective of hormone receptor status (Fig. 1F and G).^48^ Furthermore, Metabric analysis via KM plotter analyses,^49^ enabled a survival curve showing that high miR125b tumor expression is associated with increased overall survival among patients with luminal A BC (Fig. 1H). We appreciate that the analysis in Fig. 1H does not include luminal B breast cancers since the latter differs from the former primarily in having a high proliferation index and poorer prognosis. We were unable to complete similar survival analyses on miR125b2 using the KM plotter analyses program because a systematic overview of the consequences of high vs low miR125b2 expression in the BC cohort of TCGA data is currently not available with this tool. Lastly, we chose miR125b-2–3p because it is predicted to regulate genes that promote stemness.^50^

Of the miRNAs differentially upregulated in MCF-7 mammospheres exposed to AF, most are predicted to regulate apoptotic signaling pathways and DNA-binding transcription factor activator activity (Supplementary Figure 1, see online supplementary material). We have previously described the impact of AF on apoptosis and associated signaling pathways.^12,15^ Others have shown the impact of AF on DNA binding mediated via the AhR transcription factor.^51^ However, we more recently determined that AF also directly impacts luminal breast CSCs and its actions against this cell population suggest a mechanism by which AF targets stemness to confer anticancer action in TamR BC cells.^12,13^ Gene expression analysis of MCF-7 mammospheres exposed to AF for 48 h showed a 2-fold induction in miRNA expression in mammospheres relative to control (Fig. 1I). This validates the miRNA sequencing data (Supplementary Table I). Furthermore, a similar trend in induction was seen in MCF-7 monolayers albeit to a lesser extent (∼1.6 fold) than what was seen in the mammospheres (Fig. 1J), These data show that AF treatment leads to increased expression of miR125b-2–3p in luminal A BC cells.

### Mimic treatment enhanced miR125b-2-3p expression in BC cells irrespective of AhR responsiveness

To explore the roles of miR125b-2–3p in luminal BC cells, we first examined miR125b-2–3p expression in the MCF-7 cell line and MCF-7 variant cell line designated as AHR^100^. The commonly used MCF-7 cell line represents a prototypical luminal A BC subtype. The MCF-7 variant AHR^100^ cells were generated from continuous exposure to escalating concentrations of benzo[a]pyrene (BAP) until they became resistant.^43^ Their resistance to the very potent AhR agonist BAP has rendered them non-responsive to most other AhR ligands because AhR signaling mechanisms are severely impaired.^43^ The AHR^100^ cell line was chosen as an additional experimental model because AHR^100^ cells are identical to MCF-7 cells in biology except for the impaired AhR signaling activation when exposed to AhR agonists. We found that miR125b-2–3p expression is significantly lower (∼4-fold) in AHR^100^ cells compared to MCF-7 cells (Fig. 2A). We treated AHR^100^ and MCF-7 cells for 48 h with miR125b-2–3p mimic and MCF-7 cells with antagomiR125b-2–3p before analyzing miR125b-2–3p expression. Figures 2B and C show that miR125b-2–3p mimics up-regulate miR125b-2–3p expression levels (∼2- and ∼2.5-fold) in AHR^100^ and MCF-7 cells respectively. This reveals that the miR125b-2–3p mimic can induce miR125b-2–3p expression in a manner that is independent of the cell's responsiveness to AhR signaling activation. As expected, when MCF-7 cells were exposed to antagomiR-125b-2–3p, miR125b-2–3p expression was down-regulated (>2-fold) as compared to scramble control (Fig. 2D).

### miR125b-2-3p regulated stemness gene expression in luminal A BC cells and mammospheres

To determine how miR125b-2–3p regulates stemness properties in MCF-7 cells, we investigated the expression of three genes associated with stemness or oncogenicity in miR125b-2–3p mimic- and antagomiR-exposed cells. We selected *ITGA6* and *MYC* because *in silico* analysis showed predicted binding sites in their 3′UTR (untranslated region) regions for miR125b-2–3p (Supplementary Table IV, see online supplementary material).^50^*SOX2* is not a direct target of miR125b-2–3p but a single miRNA can target multiple genes, and TargetScanHuman8.0 produced a total of 4007 predicted targets of miR125b-2–3p that may include genes upstream of *SOX2* or regulators of *SOX2* (Supplementary Table V, see online supplementary material). Furthermore, *ITGA6, MYC*, and *SOX2* are established stemness genes whose transcribed proteins reprogram somatic cells to become more stem cell-like.^52–55^ Lastly, *MYC* is one of the most amplified oncogenes in BC that mediates stemness behaviors including tumor aggressiveness.^56^ We have previously characterized MCF-7 and AHR^100^ mammospheres for CSC phenotypic expression of known CSC markers.^13^ We found that MCF-7 mammospheres show increased gene expression of stemness markers *ITGA6* and *OCT4* (RT-qPCR) and increased aldehyde dehydrogenase 1 (ALDH1) protein expression (more Aldefluor™ positive stained cells) compared to monolayers.^13^ We also previously demonstrated a link between CSC biomarker *ITGA6* and BC subtype and ET resistance^12,13^*ITGA6* expression is greatest in more aggressive forms of BC stratified by subtype, while *ITGA6* expression is higher in cells made resistant to the ET agent tamoxifen. Most importantly, *ITGA6* expression is greater in BC tissues from patients treated with tamoxifen who relapsed.^12^

In the presence of the miR125b-2–3p mimic, *ITGA6* and *SOX2* expression was significantly inhibited (Fig. 2E and F) while *MYC*expression remained unchanged (data not shown). Likewise, when miR125b-2–3p was inhibited by antagomiR125b-2- 3p, *ITGA6* and *SOX2* mRNA expression levels were significantly increased in MCF-7 cells compared to scramble control (Fig. 2G and H). However, there was no significant change in *MYC* expression (data not shown). This demonstrates miR125b-2–3p inhibits stemness genes *ITGA6* and *SOX2* in luminal A BC cells. Western blot analysis revealed that when the miR125b-2–3p expression was augmented in MCF-7 and AHR^100^ cells, α6-integrin protein expression was decreased (∼2 fold) in miR125b-2–3p-treated cells compared to scramble-control (Supplementary Figure 2A and B, see online supplementary material). MCF-7 cells treated with miR125b-2–3p antagomiR demonstrated higher α6-integrin protein expression than scramble-control cells although statistical significance was not achieved (Supplementary Figure 2C).

We then evaluated whether miR125b-2–3p and AF exhibit similar effects in mammospheres to those observed in BC monolayers. As expected, AF significantly inhibited the expression of *ITGA6* and *SOX2* in the scramble-exposed mammospheres (Fig. 2I and J), but *MYC*expression remained unchanged (Fig. 2K). In DMSO-exposed mammospheres, the mimic significantly inhibited *ITGA6* but not *SOX2* nor *MYC* expression levels (Fig. 2I–K). MCF-7 mammospheres grown from miR125b-2–3p antagomiR-exposed cells showed significantly higher *ITGA6* mRNA expression compared to scramble-exposed mammospheres following exposure to DMSO (Fig. 2I). The antagomiR also reduced the ability of AF to decrease *ITGA6* expression compared to scramble-exposed (Fig. 2I). miR125b-2–3p antagomiR-exposed MCF-7 mammospheres showed significantly higher *SOX2* mRNA expression as compared to scramble-exposed mammospheres treated with DMSO (Fig. 2J). AF was able to significantly decrease *SOX2* expression levels even among mammospheres exposed to the antagomiR, suggesting that AF-mediated decrease in *SOX2* occurs in a manner that does not depend on AF's induction of miR125b-2–3p (Fig. 2J). *MYC* mRNA expression was significantly increased in MCF-7 mammospheres exposed to either DMSO or AF in the presence of the miR125b-2–3p antagomiR (Fig. 2K). This reveals that AF must induce miR125b-2–3p to suppress *MYC* expression. These data suggest that AF does not exclusively rely on miR125b-2–3p-driven mechanisms to regulate *ITGA6* and *SOX2* mRNA expression, though AF enhances miR125b-2–3p-driven inhibition of *ITGA6* and *SOX2*.

Lastly, we sought to determine whether AF-mediated α6-integrin protein suppression occurred in an AhR-dependent manner. We found AF significantly suppressed α6-integrin protein expression in MCF-7 cells, but slightly promoted α6-integrin expression in AHR^100^ cells (Supplementary Figure 3A, see online supplementary material). The AhR inhibitor αNF reversed AF-mediated α6-integrin suppression in MCF-7 cells (Supplementary Figure 3B). These data reveal that AF inhibits α6-integrin protein expression in an AhR-dependent manner.

### AF enhanced miR125b-2-3p-mediated inhibition of luminal A BC cell proliferation

**Figure 3.**
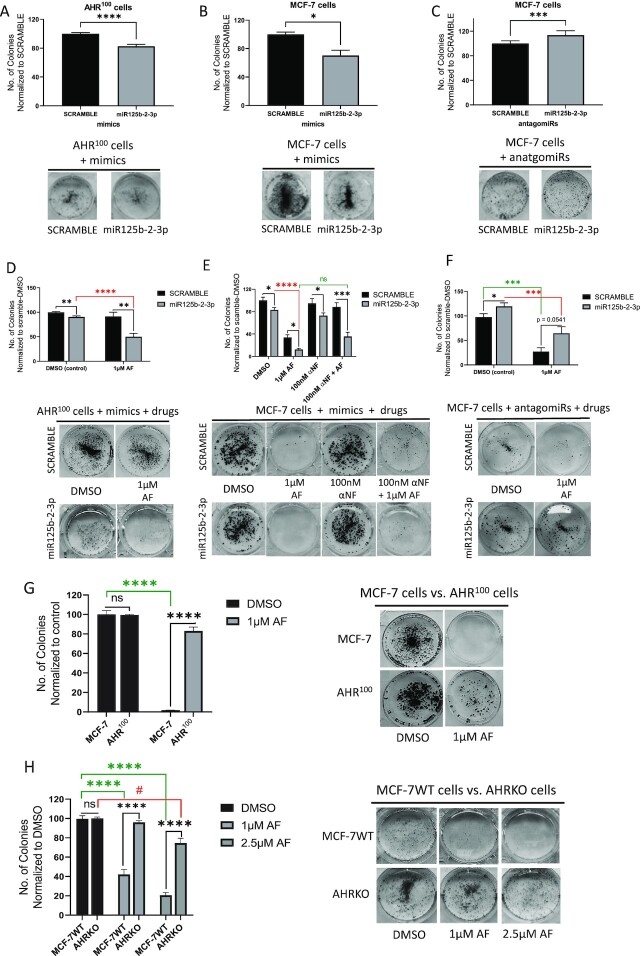
miR125b-2–3p inhibits proliferation in MCF-7 cells, while aminoflavone enhances miR125b-2–3p-mediated inhibition of proliferation. Assessment of clonogenicity of (**A**) AHR^100^ cells after exposure to miR125b-2–3p mimic vs scramble controls for 48 h, (**B**) MCF-7 cells after exposure to (**C**) miR125b-2–3p mimic or, miR125b-2–3p antagomiR vs scramble controls for 48 h, (**D**) AHR^100^ cells treated with mimics and AF, (**E**) MCF-7 cells treated with mimics, AhR-antagonist, and AF, (**F**) MCF-7 cells treated with antagomiR and AF, (**G**) MCF-7 and AHR^100^cells treated with AF, (**H**) MCF-7WT and AHRKO cells treated with AF. The colony numbers were counted and normalized to control. ^#^*P* < 0.05, ^****^P < 0.0001, ***P < 0.001, **P < 0.01, *P < 0.05 and ns indicates not significant compared to scramble. Columns, mean of three independent experiments: error bars, SEM.

Proliferation or clonogenicity is an important stemness characteristic that promotes breast tumor growth.^57^ We used the colony-forming assay in MCF-7 and AHR^100^ cells treated with either the miR125b-2 mimic or the antagomiR125b-2–3p to ascertain miR125b-2–3p's role in luminal A BC cell proliferation. Fewer colonies formed in AHR^100^ and MCF-7 cells following treatment with miR125b-2–3p mimic relative to scramble (Fig. 3A and B). Interestingly, AHR^100^ cells displayed an even greater decrease in clonogenicity following treatment with mimic relative to scramble (Fig. 3A). MCF-7 cells formed more colonies when treated with antagomiR125b-2–3p as compared to scramble (Fig. 3C). These data show that miR125b-2–3p suppresses proliferation in luminal BC cells irrespective of their responsiveness to AhR agonists.

In cells co-treated with AF and the miR125b-2–3p mimic, AF significantly enhanced the sensitivity of AHR^100^ cells to the miR125b-2–3p mimic (Fig. 3D). Additionally, the trend from Fig. 3A was repeated; miR125b-2–3p decreased proliferation in DMSO-treated AHR^100^ cells (Fig. 3D). Importantly, MCF-7 cells co-treated with miR125b-2–3p mimic and AF resulted in fewer colonies as compared to treatment with the mimic alone (Fig. 3E). Also, the trend from Fig. 3B was repeated; miR125b-2–3p decreased proliferation in DMSO-treated MCF-7 cells (Fig. 3E). We then selected the AhR inhibitor, αNF, to test if blocking AhR-activation would affect sensitivity to AF. We initially selected αNF because it is non-toxic to cells at effective doses.^58^ We observed that αNF successfully blocked AF from inhibiting proliferation in scramble-treated MCF-7 cells. However, despite the presence of αNF, the mimic-exposed MCF-7 cells still showed sensitivity to AF compared to scramble-exposed cells cotreated with αNF (Fig. 3E). There was also no significant difference between proliferation in mimic-exposed cells treated with AF alone and mimic-exposed cells treated with both AF and αNF (Fig. 3E). Lastly, cells co-treated with AF and the miR125b-2–3p antagomiR produced significantly more colonies as compared to those treated with the antagomiR alone, while antagomiR125b-2–3p again led to production of more colonies in DMSO-exposed MCF-7 cells (Fig. 3F). These data suggest that miR125b-2–3p inhibits proliferation in MCF-7 cells and AF enhances miR125b-2–3p-mediated anti-proliferation in a manner that partially relies on AhR.

When MCF-7 and AHR^100^ cells were treated with 1 µM AF alone, AHR^100^ cells produced more colonies than MCF-7 cells but there was no significant decrease compared to AHR^100^ cells treated with DMSO (Fig. 3G). Similarly, when MCF-7WT and AHRKO cells were exposed to two different concentrations of AF, WT cells produced far fewer colonies after AF treatment than the AHRKO cells (Fig. 3H). Interestingly, AHRKO cells showed a slight response to the higher 2.5 µM concentration, which suggests that at higher concentrations AF may adopt non-AhR-mediated mechanisms to inhibit proliferation.

### mir125b-2-3p decreased the number of mammospheres derived from AhR-responsive cells, and AF upregulated miR125b-2-3p to reduce mammosphere number

**Figure 4.**
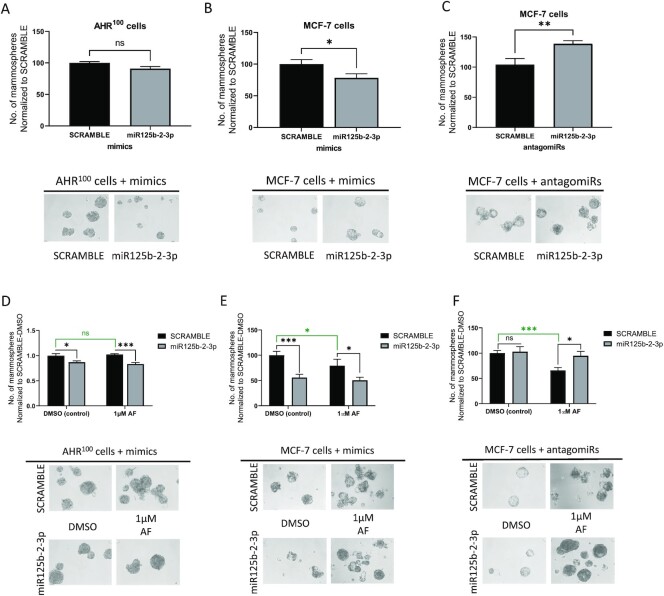
miR125b-2–3p inhibits mammosphere formation in breast cancer cells. Assessment of mammosphere formation frequency in (**A**) AHR^100^ cells after miR125b-2–3p mimic or scramble treatment, (**B**) MCF-7 cells after miR125b-2–3p mimic or scramble treatment, (**C**) MCF-7 cells after miR125b-2–3p antagomir or scramble treatment, (**D**) 48 h AF treatment of AHR100 cells in the presence or absence of miR125b-2–3p mimic, (**E**) 48 h AF treatment of MCF-7 cells in the presence or absence of miR125b-2–3p mimic, and (**F**) 48 h AF treatment of MCF-7 cells in the presence or absence of miR125b-2–3p antagomir. Mammospheres were cultured for 5 days before requisite treatments. Images of representative mammospheres at 40x magnification. ***P < 0.001, **P < 0.01, *P < 0.05 relative to control. ns indicates not significant. Columns, mean of three independent experiments: error bars, SEM.

We used the mammosphere formation assay to evaluate CSC potential in BC cells. The mammosphere formation assay is ideal because mammospheres enrich for CSCs.^13,59^ We have previously shown that the CD44^+^/CD24^−/low^ cell population is increased from 0.02% in the untreated MCF-7 monolayer population to 0.1% in the untreated MCF-7 mammosphere population. Importantly, we also showed that while tamoxifen further expanded the CD44^+^/CD24^−/low^ population to 0.32% in MCF-7 mammospheres, AF reduced the CSC population back to 0.02%, which is the same proportion as that found within untreated MCF-7 monolayers.^13^ In this study, we sought to first determine whether miR125b-2–3p regulates CSC potential in MCF-7 cells and whether AF must induce miR125b-2–3p to decrease mammospheres derived from luminal BC cells. The miR125b-2–3p mimic was unable to significantly reduce mammosphere numbers in AHR^100^ cells (Fig. 4A) but the miR125b-2–3p mimic decreased the number of MCF-7 mammospheres (Fig. 4B). Conversely, exposure of MCF-7 mammospheres to the miR125b-2–3p antagomiR increased mammosphere production relative to scramble control (Fig. 4C).

Surprisingly, the miR125b-2–3p mimic significantly decreased mammosphere formation in DMSO-treated AHR^100^ mammospheres, and AF (1 µM) significantly enhanced the ability of the miR125b-2–3p-mimic to disrupt AHR^100^ mammospheres (Fig. 4D). This shows that AF can reduce mammosphere numbers even in AhR unresponsive cells. Significantly fewer mammospheres were present in miR125b-2–3p-mimic-exposed MCF-7 cells as compared to scramble-exposed MCF-7 cells following DMSO treatment (Fig. 4E). Treatment of cells with AF and the miR125b-2–3p mimic led to a greater reduction in mammosphere number than treatment with AF alone (Fig. 4E). AF exhibited a reduced capacity to disrupt mammospheres exposed to the miR125b-2–3p-antagomiR as compared to scramble-exposed mammospheres. AntagomiR125b-2–3p produced more mammospheres after DMSO exposure though it did not quite reach statistical significance as with mimic-only treated mammospheres (Fig. 4F). This suggests AF suppresses CSC potential once it up-regulates miR125b-2–3p. Taken together, the data show that miR125b-2–3p regulates CSC potential in luminal BC cells that are AhR-responsive, and that AF reduces CSC potential once it induces miR125b-2–3p via an AhR-independent mechanism.

### miR125b-2-3p inhibited migration in MCF-7 cells and AF enhanced miR125b-2-3p-mediated inhibition of migration in cells irrespective of AhR responsiveness

Migration, invasion and EMT are stemness properties that enable cancer cells to metastasize and promote relapse in patients. Using the wound healing assay, we sought to determine whether miR125b-2–3p regulates migration in luminal BC cells. Migration rates for each AF treatment were compared to control treatment (0.01% DMSO), and the mean migration rate for DMSO (0 µm/h) is represented by the *x*-axis. Negative values denote a slower migration rate relative to DMSO and positive values denote a faster migration rate relative to DMSO.

**Figure 5.**
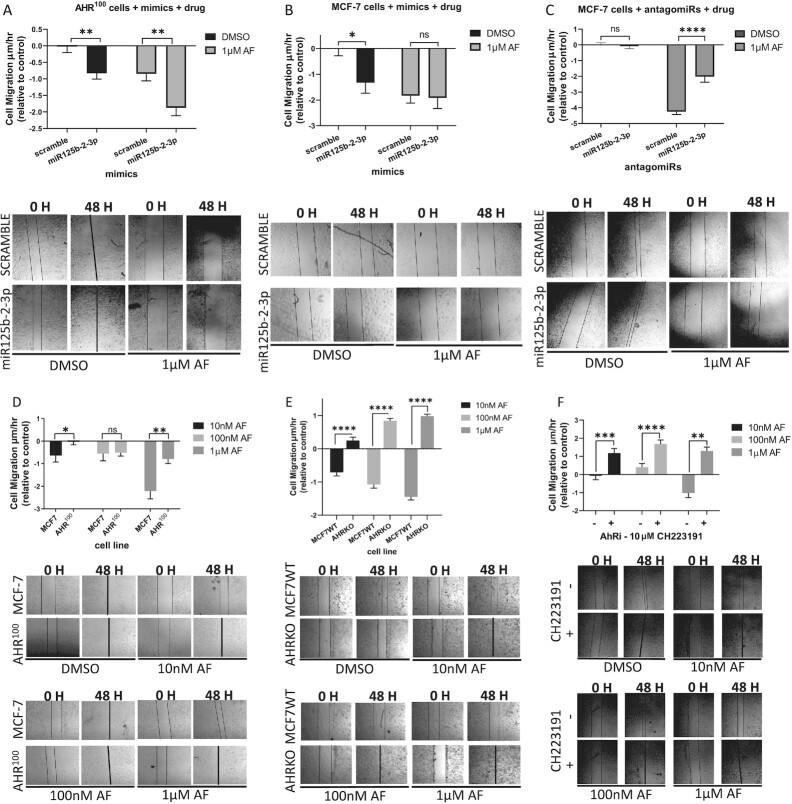
AF enhances miR125b-2–3p-mediated migration of breast cancer cells. Migration assay analysis of AHR^100^ and MCF-7 cells treated with either miR125b-2–3p mimics or antagomiRs vs their scramble controls for 48 h. The width of the gap between two patches of cells was measured, migration rate was calculated and any significant differences between the control group and treatment group were identified. Migration assay analysis of (**A**) AHR^100^ cells exposed to either miR125b-2–3p mimic or scramble then with AF or DMSO, (**B**) MCF-7 cells exposed to either miR125b-2–3p mimic or scramble then with AF or DMSO, (**C**) MCF-7 cells exposed to either miR125b-2–3p antagomiR or scramble then AF or DMSO, (**D**) MCF-7 and AHR^100^ cells exposed to either AF or DMSO, (**E**) MCF-7WT and AHRKO cells exposed to either AF or DMSO, and (**F**) MCF-7 cells exposed to either miR125b-2–3p antagomiR or scramble then AF or DMSO treated with AhR-antagonist CH223191 then AF or DMSO. ^****^P < 0.0001, ***P < 0.001, **P < 0.01, *P < 0.05, compared to DMSO and ns indicates not significant. Columns, mean of three independent experiments: error bars, SEM.

We observed significantly slower migration rates in both non-treated AHR^100^ and MCF-7 cells exposed to miR125b-2–3p mimics compared to scramble (Fig. 5A and B). In AHR^100^ cells, miR125b-2–3p-mediated migration suppression was enhanced with AF (1 µM) treatment (Fig. 5A). While the mimic alone impeded MCF-7 cell migration, AF only slightly and insignificantly enhanced its anti-migration properties (Fig. 5B), suggesting that AF suppresses migration via AhR-independent mechanisms following its upregulation of miR125b-2–3p. When miR125b-2–3p was inhibited in MCF-7 cells no statistically significant change to migration rate was observed (Fig. 5C), which suggests additional regulators of migration play a role when miR125b-2–3p is inhibited. On the other hand, the antagomiR significantly counteracted the ability of AF (1 µM) to inhibit the migration of MCF-7 cells (Fig. 5C). Taken together, the data show that miR125b-2–3p is a key regulator of migration in luminal A BC, which AF enhances in an AhR-independent manner, however additional regulators are also involved in the absence of drug treatment.

To further delineate the role of AhR in AF-mediated suppression of cell migration, we evaluated migration in MCF-7, AHR^100^ and AHRKO cells at varying concentrations. We found that AF (10 nM and 1 µM) inhibited migration significantly better in MCF-7 as compared to AhR^100^ cells (Fig. 5D). Similarly, AF inhibited migration in MCF-7WT cells but promoted migration in AHRKO cells at all three concentrations (Fig. 5E). We also treated MCF-7 cells with a selective and potent AhR antagonist, 2-methyl-2H-pyrazole-3-carboxylic acid (2-methyl-4-o-tolylazo-phenyl)-amide (CH223191), along with AF. CH223191 is a bona fide AhR antagonist, unlike αNF, which can be competitively excluded by stronger agonists and antagonists.^60^ Like the AHRKO cells, MCF-7 cell migration was promoted rather than inhibited in the presence of CH223191, with the greatest shift observed at 100 nM AF (Fig. 5F). Interestingly, CH223191 treatment alone slightly increased MCF-7 cell migration relative to DMSO control (Supplementary Figure 3), which may have contributed to the increased migration rates observed with AF and CH223191 co-treatment (Fig. 5F). Taken together, this suggests that when the AhR is absent or pharmacologically inhibited, AF may bind different receptors to activate migration-promoting mechanisms in BC cells.

## Discussion

miRNAs regulate gene expression post-transcriptionally and are often dysregulated in human tumors.^20^ They can function as oncogenes or tumor suppressors. In the present study, we used miRNA sequencing to profile miRNAs in MCF-7 mammospheres exposed to AF or 0.1% DMSO. We identified 206 significant DEmiRNAs (*P* adjusted < 0.01 and |log2 (fold-change)| > 0.5). We found that AF significantly up-regulated miR125b-2–3p and several other miR125 family members. We then validated the ability of AF to induce miR125b-2–3p expression in MCF-7 mammospheres and monolayers. Induction was lower in monolayers, which was anticipated since mammospheres enrich for CSCs and this miRNA impacts breast CSC self-renewal properties. AF upregulates miR125b-2–3p to promote its anticancer and CSC disrupting actions. It is also possible that this miRNA's expression is dependent on the cellular environment that mammospheres more accurately represent than the 2D monolayers. Furthermore, our discovery that AHR^100^ cells exhibit low miR125b-2–3p expression relative to MCF-7 wild-type cells suggests a functional link between the AhR and miR125b-2–3p. While much of our study was performed in luminal A MCF-7 cells and MCF-7 cell line variants, it is important to appreciate that miR125b2-3p actions can be applied to luminal BC in general since it has been shown that miR125b-2 expression is down-regulated in ER + luminal BC tissues compared to normal breast tissues^41,42^ Furthermore, we showed that high tumor expression of another miR125 family member—miR125b—correlates with increased overall survival in ER + HER2- (luminal A) BC (Fig. 1D).^49^ Luminal B BC differs from luminal A BC in part by expressing a higher proliferative index and lower patient survival rates.^61^ Lastly, we showed that miR125b-2 expression is also very low in BC tissues vs normal tissues irrespective of hormone receptor status, while miR125b-2–3p's expression is very low in BC tissues without stratification by subtype compared to normal tissue. Stratification by subtype analysis was not possible for miR125b-2–3p because this feature is not yet available using mir-tv.^47^ We also confirmed the TCGA data with in-house q-PCR analysis of 5 BC tissue FFPE samples. We showed that miR125b-2–3p expression is lower in BC tissues compared to non-tumorigenic MCF-10A breast epithelial cells. Comparison with normal-adjacent tissue from the same patients is ideal but such samples were unavailable to us so the MCF-10A cells were used. The significantly low expression of miR125b family members, and miR125b-2–3p specifically, in patient BC tissue may suggest that this miRNA is tumor-suppressive in the more dangerous BC subtypes as well. However, this miRNA's actions must be empirically evaluated in additional BC subtypes in future studies.

We showed that miR125b-2–3p regulates stemness gene and protein expression in luminal A BC cells and mammospheres. Increased expression of miR125b-2–3p inhibited the expression of at least two stemness genes in MCF-7 cells. Decreased miR125b-2–3p expression resulted in increased stemness gene expression in these same cells (except for *MYC*) and mammospheres. Furthermore, in MCF-7 mammospheres, miR125b-2–3p suppression reduced AF's ability to inhibit mRNA expression of stemness genes while augmentation of miR125b-2–3p slightly enhanced AF's ability to suppress *ITGA6*. Our data suggest that in mammospheres, AF partially depends on miR125b-2–3p to regulate *ITGA6* and *SOX2* expression. On the other hand, our data suggest that AF inhibits *MYC* via miR125b-2-3p upregulation. miR125b-2–3p may directly regulate *ITGA6* and *MYC* and may regulate *ITGA6* and *SOX2* expression indirectly by targeting genes upstream of *ITGA6* and *SOX2*. Western blot data showed that miR125b-2–3p induction leads to decreased α6-integrin protein expression in MCF-7 and AHR^100^ cells. AF treatment also decreased α6-integrin expression in MCF-7 cells but not in AHR^100^ cells. The AhR antagonist reversed AF-mediated suppression of α6-integrin expression in MCF-7 cells to indicate that when AhR-signaling mechanisms are impaired, AhR-independent signaling mechanisms are activated by AF to suppress α6-integrin expression. We previously demonstrated that AhR-unresponsive cells impeded AF-mediated suppression of α6-integrin expression and disruption of mammospheres.^13^

Next, our data suggest that miR125b-2–3p functions as a tumor-suppressor since it not only diminishes CSC activity but thwarts migration and proliferation in a luminal A BC cell line and two variants. While miR125b-2–3p inhibits proliferation, mammosphere production, and migration in MCF-7 cells, it also confers these actions in AHR^100^ and AHRKO cells but to a lesser extent. It is noteworthy that AF enhanced miR125b-2–3p-mediated inhibition of proliferation in MCF-7 cells even in the presence of an AhR antagonist. Additionally, AHR^100^ cells, which were initially resistant to AF became sensitive to AF when miR125b-2–3p was augmented. AHRKO cells also showed slight sensitivity to a higher AF concentration. This suggests that when the AhR is absent, blocked, or defective, AF activates other signaling mechanisms to inhibit proliferation.

We showed that 1 µM AF enhanced miR125b-2–3p-mediated inhibition of migration. In AHR^100^ cells, 1 µM AF significantly slowed migration compared to scramble-exposed cells. In antagomiR-treated MCF-7 cells, AF was less able to inhibit migration, while the presence of mimic slightly increased AF's antimigration activity. Inhibiting AF-mediated AhR signaling activation in MCF-7 cells significantly accelerated migration and similarly, migration was accelerated in AHRKO cells by AF treatment. Taken together, these data suggest that when the AhR is defective, a higher AF concentration is needed to inhibit migration. When AhR is completely knocked out, or blocked by an antagonist, AF may activate alternate signaling mechanisms that promote migration in luminal BC cells.

We also showed that miR125b-2–3p reduces CSC activity in luminal A BC cells. MCF-7 cells produced fewer mammospheres when exposed to mimic and MCF-7 cells produced more mammospheres when exposed to antagomiR125b-2–3p. AHR^100^ cells showed a slight decrease in mammosphere formation with mimic treatment, which initially suggested that AhR status may play a role in miR125b-2–3p-mediated CSC regulation. However, when we proceeded with drug treatment in mimic-exposed mammospheres, we observed fewer AHR^100^ mammospheres after DMSO treatment relative to scramble. This suggests that miR125b-2–3p can confer anti-CSC actions on AhR-deficient cells. Upon consideration of our size-exclusion criteria for mammosphere counting it is plausible that statistical significance was not reached in non-treated AHR^100^ mammospheres relative to scramble (Fig. 4A) because small mammospheres that still met the size criteria were included in the count. We encountered a similar issue after AF treatment when counting MCF-7 mammospheres grown from scramble-treated cells (Fig. 4E). We also showed that antagomiR treatment significantly increased CSC activity as shown by greater mammosphere production (Fig. 4C), but while the trend persisted, statistical significance was absent in DMSO-exposed mammospheres (Fig. 4F). It is quite possible that mammosphere size again played a role in our count. In fact, we noticed a pattern of decreased statistical significance in our DMSO-treated assay conditions after initial exposure to either mimic or antagomiR. This suggests two more possibilities. First, the additional steps required to move our mimic- or antagomiR-treated cells on to additional assays and treatments may have affected their behavior. Second, the recommended exposure time for the AUM-FANA transfection system is 48–72 h from the beginning to the end of the experiment. Our proliferation, migration, and mammosphere formation assays all last longer than 72 h, which also may have affected behavior. However, even with these considerations, taken together, the data suggest that miR125b-2–3p inhibits CSC activity irrespective of AhR status, such that other transcription factors aside from AhR promote its expression and anti-CSC actions.

Estrogen receptor (ER) and AhR signaling crosstalk can have inhibitory effects on AhR ligand and ER ligand activity, as reviewed extensively by Matthews and Gustafsson.^62^ The ER and the AhR are ligand-activated transcription factors that upon ligand binding, lead to receptor recruitment to either estrogen-response elements or xenobiotic-response elements of target genes.^62^ Matthews *et al*. also showed that when the AhR is active, it can redirect ER from ER target genes to bind AhR target genes.^63^ This suggests that the AhR can regulate the ER as well as estrogenic responses in BC cells. It is also possible that the ER can regulate AhR and xenobiotic responses in BC cells. This may help to explain why AF is so effective in MCF-7 cells and mammospheres but also why at times AF produces effects in the AhR-active cells in the presence of an AhR antagonist, or in AhR-defective AHR^100^ cells and in AHRKO cells.

The list of 4007 predicted targets (Supplementary Table V) from TargetScanHuman8.0^50^ includes the ER genes *ESR1* and *ESR2*, which are involved in ER signaling, as well as two AhR signaling genes *ARNT1* and *ARNT2* that could prove to be bona fide targets in future studies. The proteins that these genes transcribe (ERα, ERβ, and ARNT) may play roles in AhR signaling that may affect how AF behaves when the AhR is blocked or impaired. Evidence suggests a benefit of the anti-cancer actions of AhR ligands via various signaling mechanisms. AF induces single-strand DNA breaks and forms DNA–protein cross-links dependent on histone H2AX phosphorylation in BC cells.^64,65^ We previously showed that AF suppresses α6-integrin-Src-Akt signaling in breast cancer cells.^12^ TargetScanHuman8.0 also predicted *ERRB2* as a miR125b-2–3p target. Several studies have found that miR125b acts through the ErbB2/Her2 pathway to regulate cell survival, proliferation, migration, EMT, and drug resistance in BC^32,34^,^66–68^ Our data suggest that AF promotes the recruitment of AhR to the miR125b-2–3p promoter which negatively regulates ER and Her2 signaling and inhibits the expression of stemness genes to thwart migration, colony formation, and mammosphere formation in luminal A BC cells. Understanding miRNAs, their targets, and the underlying mechanisms that the target genes use to drive disease progression are vital to developing more effective therapies for BC patients. MCF-7 represents luminal A BC, which is less aggressive and associated with a good prognosis. Therefore, it is worth examining in future studies, whether AF and miR125b-2–3p show similar effects on representative cell lines to other BC subtypes like luminal B and the very aggressive triple negative subtype, which are associated with poor prognoses. These data provide proof of concept to lay the groundwork for such future studies. Furthermore, the functional connections between the AhR and miR125b-2–3p and *ITGA6* must be further explored to fully establish whether a miR125b-2–3p-driven mechanism of regulating stemness through *ITGA6* truly exists.

## Conclusions

Our findings suggest a tumor-suppressive role for miR125b-2–3p in luminal A BC. Furthermore, we show that miR125b-2–3p inhibits mRNA expression of stemness genes in BC cells. miR125b-2–3p also suppresses proliferation, migration, and CSC-potential in MCF-7 cells. AF increases miR125b-2–3p expression in MCF-7 cells and mammospheres to inhibit *ITGA6* and *SOX2* expression and disrupt CSC-driven behaviors in BC cells. Though the data suggest that AF upregulates miR125b-2–3p in luminal BC to confer its anticancer and stem cell suppressing actions, the relationship between miRNAs, target genes, and signaling mechanisms remains complex and eludes our complete understanding. Often the interplay between these mediators is dependent on the cellular context and the tumor microenvironment. This study provides a rationale for the further development of anticancer AhR ligands for use in combination with ET or other targeted therapy agents to combat ET-resistant luminal BC. Furthermore, because CSCs are crucial drivers of drug resistance and disease progression, this study provides a basis for identifying anticancer agents that may target CSCs via AhR signaling activation more effectively than conventional therapies.

## Supplementary Material

pbac008_Supplemental_Figures_and_Tables
