# Supplementary material for: Aminoflavone upregulates putative tumor suppressor miR-125b-2-3p to inhibit luminal A breast cancer stem cell-like properties
Source: Precis Clin Med. 2022 Mar 28;5(2):pbac008. doi: 10.1093/pcmedi/pbac008 (PMC9172653; doi:10.1093/pcmedi/pbac008)

Supplementary Figure 1

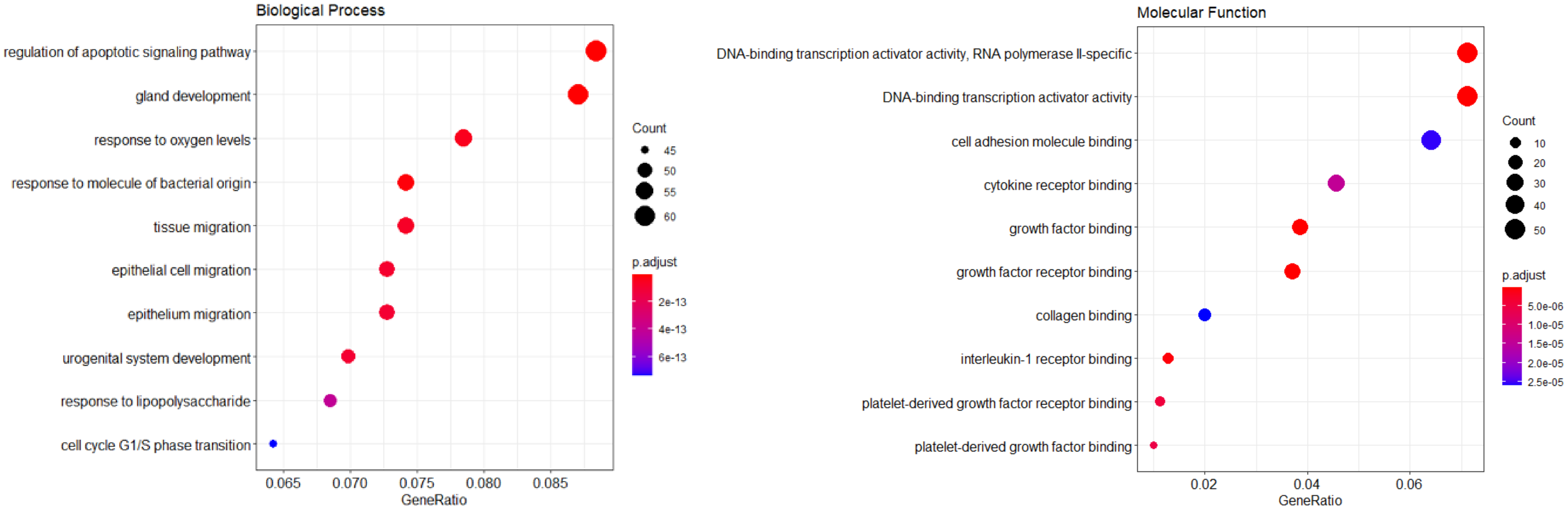

Supplementary Figure 2

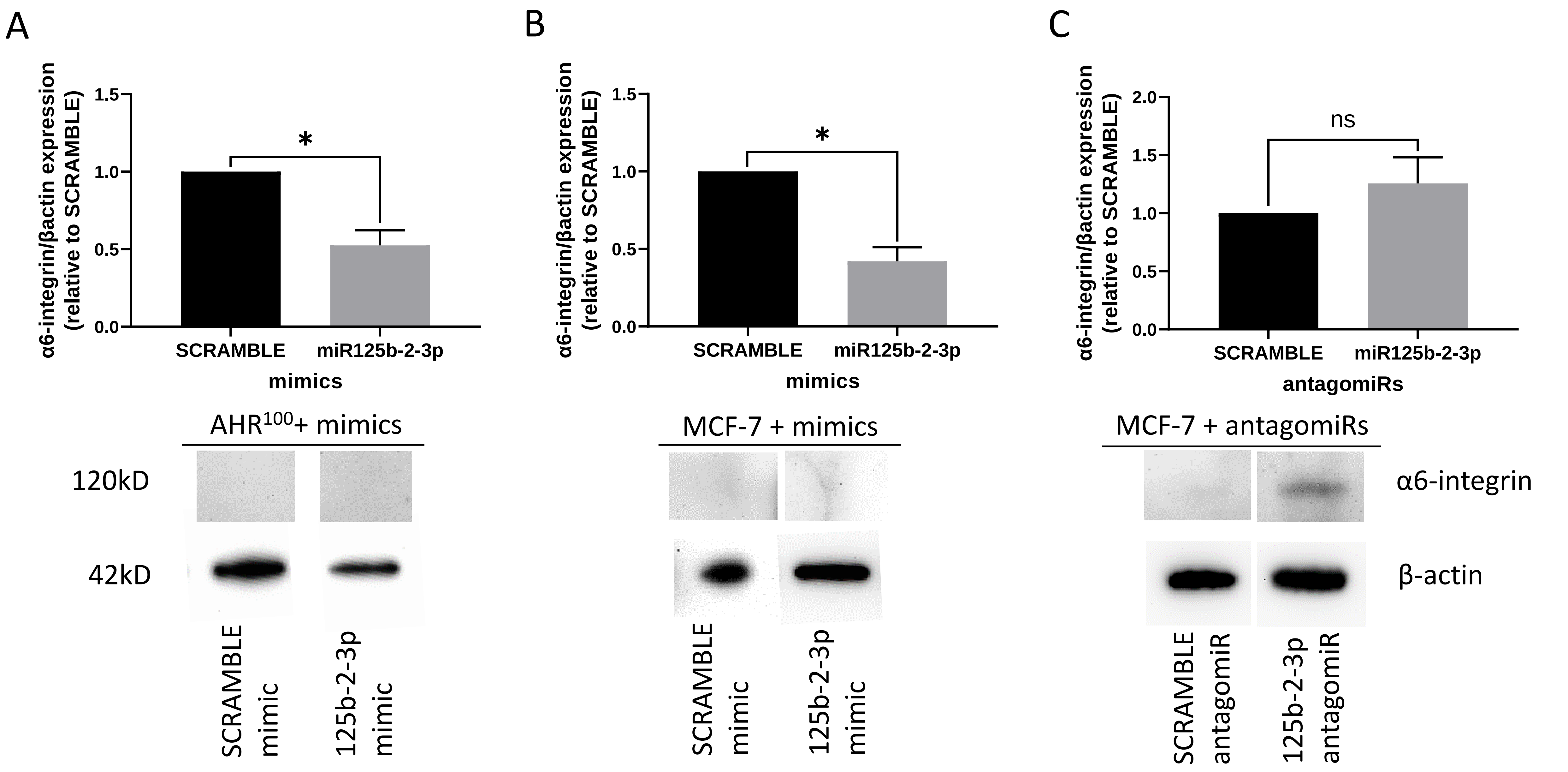

Supplementary Figure 3

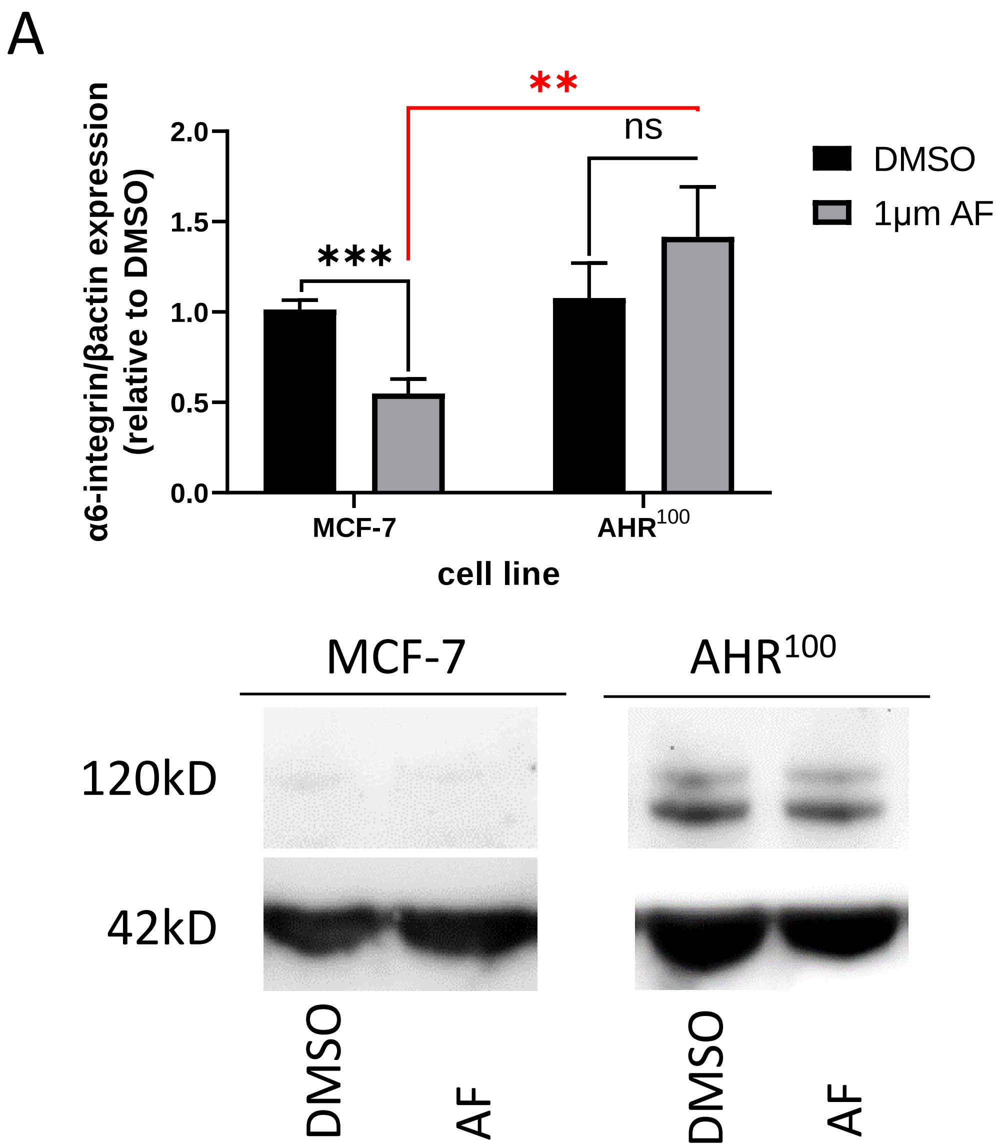

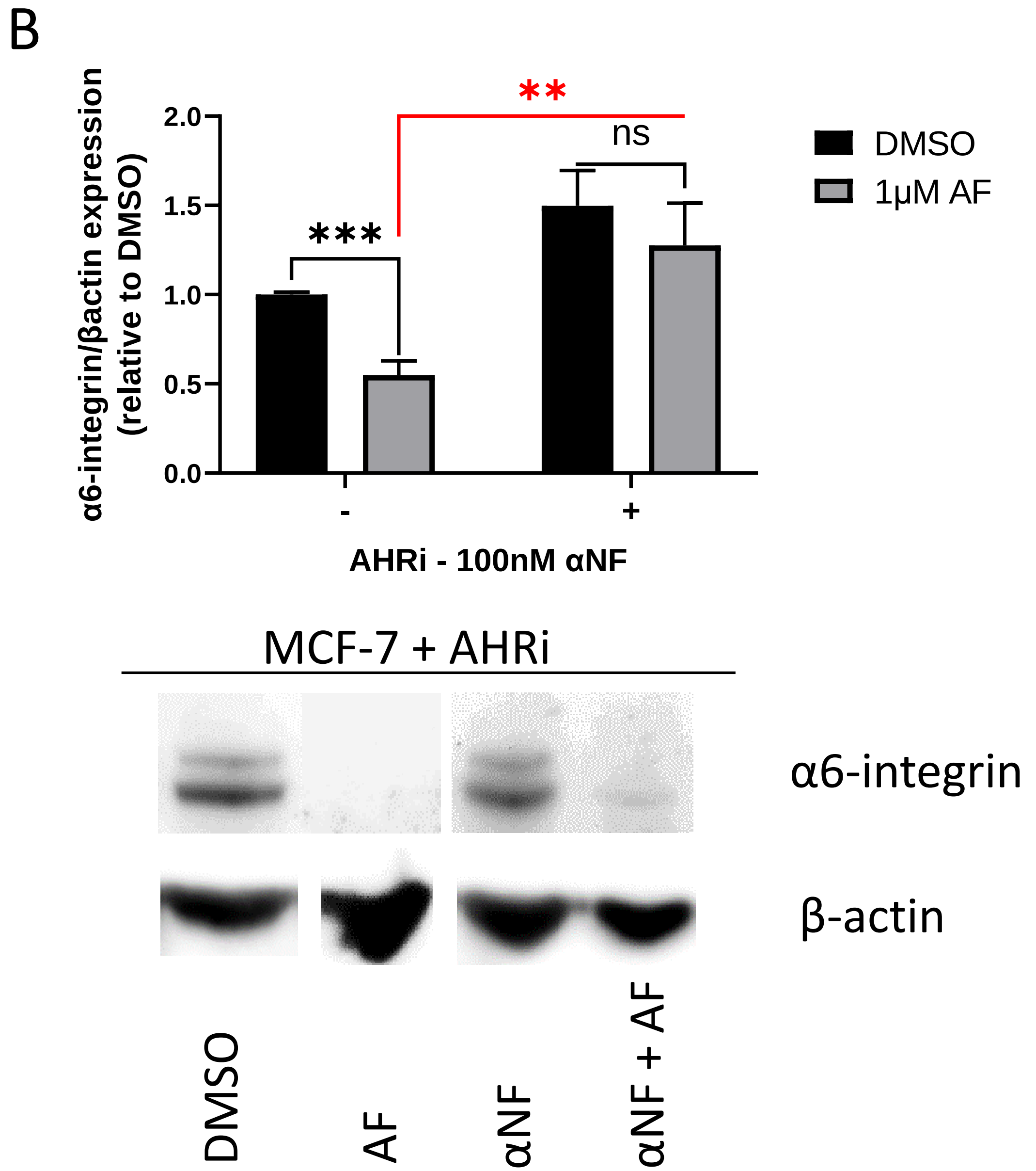

Supplementary Figure 4

MCF-7 cells after exposure to CH223191 or DMSO alone

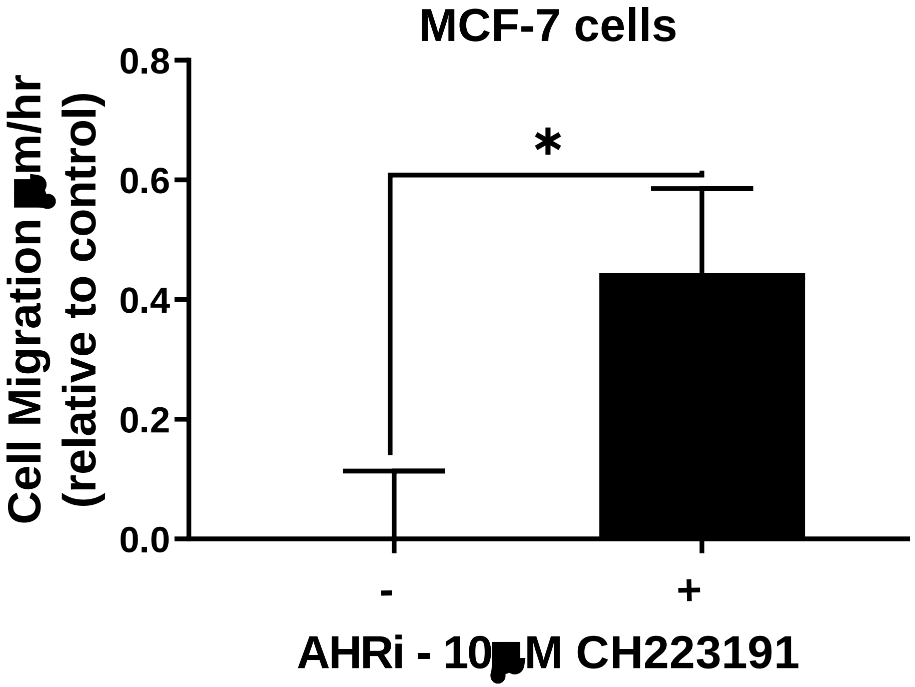

Supplement: pbac008_Supplemental_Figures_and_Tables [file pbac008_supplemental_figures_and_tables.zip › Supplementary Figures 1-4.pdf]
